# Diet in Acne

**Published:** 1899-03

**Authors:** 


### Diet in Acne.

The regulation of the diet in this troublesome and so often obstinate affection, is now generally admitted to be the most important element in the treatment of the disease. Patients themselves will usually have been trying various dietary experiments along with the ordinary home remedies before consulting a physician. Unless, however, the most explicit directions are given as to the proper diet, serious mistakes will be made by patients in the selection of foods, and especially as to its quantity. As Dr. Jackson says, in his Manual of Diseases of the Skin,* "The well-to-do are prone to eat too much, and it is remarkable how rapidly their acne will improve by

*From advance sheets of the third edition of a Ready-Reference Hand-Book of Skin Diseases, by George Thomas Jackson, M. D. Lea Brothers & Co., Publishers.

reducing their diet to the simplest elements. In many of them a milk diet, provided milk agrees with them, will accomplish a marked benefit." On the other hand, many young girls almost starve themselves entertaining the mistaken idea that a low diet will give them a fine complexion. Nothing could well be less true than this. Especially is there a prejudice against butter. The old explanation that skin eruptions were mainly due to the use of too much butter still remains absolutely true for most non-medical people, and even for some medical men. That butter should be used freely and that codliver oil and iron should be the only drugs required in many cases, as Dr. Jackson insists, would, to these good old conservatives, seem rank heresy. It is evident that more definite ideas as to the diathesis that underlies the etiology of acne have been acquired, and that the dietetic management of it rather than any empiric use of vaunted specifics constitutes the most modern therapeusis of this extremely frequent and bothersome condition.

